# Early CytoSorb Hemoadsorption in a Neutropenic Acute Myeloid Leukemia Patient with Carbapenem-Resistant *Pseudomonas* Septic Shock and ARDS

**DOI:** 10.3390/diseases13120382

**Published:** 2025-11-24

**Authors:** Wei-Hung Chang, Ting-Yu Hu, Li-Kuo Kuo

**Affiliations:** 1 Department of Critical Care Medicine, MacKay Memorial Hospital, Taipei 10449, Taiwan; peacejaycool@gmail.com (W.-H.C.); lmn4093@gmail.com (L.-K.K.); 2 Department of Medicine, Mackay Medical College, New Taipei City 25245, Taiwan

**Keywords:** neutropenic sepsis, acute myeloid leukemia, carbapenem-resistant *P. aeruginosa*, antimicrobial resistance, ARDS, CytoSorb, septic shock, adjunctive therapy

## Abstract

Neutropenic patients with acute myeloid leukemia (AML) are at high risk for severe, multidrug-resistant infections. Sepsis due to carbapenem-resistant *Pseudomonas aeruginosa* (CRPA) in this population often leads to septic shock and acute respiratory distress syndrome (ARDS), with historically poor outcomes. CytoSorb™ hemoadsorption has been proposed as an adjunctive therapy for refractory septic shock, but evidence in hematologic malignancies remains limited. This report describes a 29-year-old male with newly diagnosed AML complicated by neutropenic fever, bacteremia due to CRPA, and subsequent hospital-acquired pneumonia progressing to ARDS. Despite multiple antibiotic regimens and aggressive intensive care management, including mechanical ventilation, prone positioning, and continuous renal replacement therapy (CRRT), the patient developed refractory septic shock with persistent lactic acidosis and elevated inflammatory markers. Early adjunctive CytoSorb hemoadsorption was initiated, guided by maximal CytoScore criteria, as part of a comprehensive supportive strategy. Following CytoSorb therapy, the patient demonstrated transient hemodynamic and biochemical improvement; however, profound neutropenia and multi-organ failure persisted. Microbiological clearance of CRPA was not achieved; given confirmed colistin susceptibility and unknown carbapenemase mechanism, a salvage combination of colistin plus ceftazidime–avibactam was employed. Transient hemodynamic improvement was observed after CytoSorb initiation; however, cytokine assays were not performed, and microbiological clearance was not achieved, precluding any mechanistic attribution to CytoSorb. This case highlights the complexity of managing CRPA sepsis and ARDS in neutropenic AML patients, and the challenges in attributing observed clinical improvement to CytoSorb therapy in the context of multiple simultaneous interventions. The absence of cytokine assays (e.g., IL-6, TNF-α) precludes any mechanistic attribution of observed changes to cytokine adsorption, and interpretation should remain descriptive rather than causal. Observed transient changes occurred amid simultaneous interventions (broad-spectrum antibiotics, CRRT, prone ventilation, corticosteroids, and filgrastim), precluding attribution to any single therapy, including CytoSorb. Given the fatal outcome and persistent CRPA positivity, the clinical impact of this observation is limited, and the generalizability of a single-case report is restricted. Cautious interpretation is warranted, and CytoSorb may be considered as part of a comprehensive care bundle rather than as a standalone solution. Alternative tetracycline-based combinations were reviewed but not adopted under our center’s salvage protocol for this XDR presentation. Future studies are warranted to clarify its clinical benefit and optimal timing in this population.

## 1. Introduction

Acute myeloid leukemia (AML) confers profound immunosuppression, predisposing patients to life-threatening infections during chemotherapy-induced neutropenia [1].

Among these infections, carbapenem-resistant *P. aeruginosa* (CRPA) has emerged as a major therapeutic challenge due to limited antibiotic options and high mortality in immunocompromised hosts [2].

Management of CRPA sepsis in neutropenic patients remains extremely challenging [3].

While prompt antibiotic administration, organ support, and source control remain the cornerstones of therapy [4], the emergence of extensively drug-resistant strains often necessitates individualized salvage regimens such as colistin-based combinations, for which evidence remains limited in this population [5].

CytoSorb™ hemoadsorption has been explored as an adjunctive therapy in refractory septic shock [6], but evidence in hematologic malignancies is scarce [7].

Early initiation guided by severity scoring (e.g., CytoScore) has been proposed to optimize timing, though clinical benefit remains uncertain [8].

Here, we describe a fatal case of CRPA septic shock and ARDS in a neutropenic AML patient, emphasizing the difficulties of infection eradication, the transient response to CytoSorb therapy, and the challenges in distinguishing device effect from concurrent interventions.

## 2. Case Presentation

### 2.1. Patient Background

A 29-year-old male with newly diagnosed acute myeloid leukemia (AML), cytogenetically 45,XY,inv(3)(q21q26.2),-7, presented with a complex medical history including prior right nasal alar cellulitis (associated with *P. aeruginosa*), postoperative peritonitis with abdominal surgery, and multiple episodes of neutropenic fever.

### 2.2. Admission and Chemotherapy

The patient was admitted for chemotherapy and developed febrile neutropenia complicated by CRPA bacteremia, which progressed to septic shock and ARDS despite broad-spectrum antimicrobial therapy. Detailed chemotherapy regimens are provided in Supplementary Table S1.

### 2.3. ICU Course and ARDS

**Figure 1:**
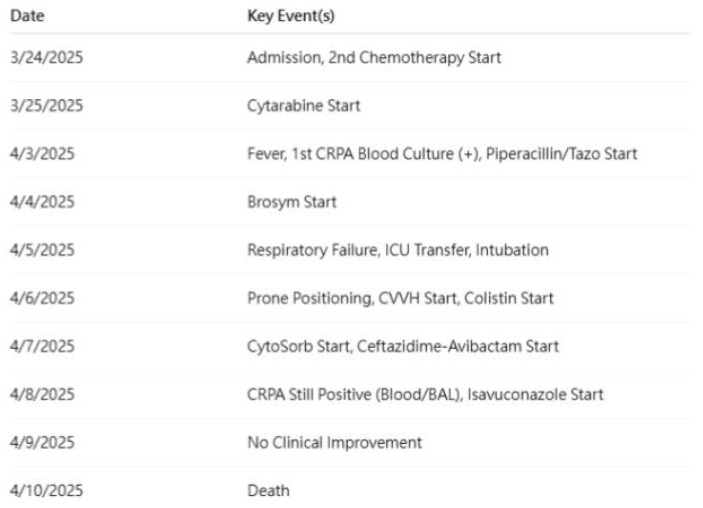
Timeline of key clinical events and interventions. Key events include onset of fever, first positive CRPA culture, escalation of antibiotics, ICU admission, intubation, initiation of CVVH and CytoSorb™, and clinical deterioration/death. Note: Timeline constructed from daily clinical records and laboratory results. Note: Multiple co-interventions overlap temporally with Cystospore initiation; the timeline is intended to visualize confounding rather than imply causality.

**Figure 2:**
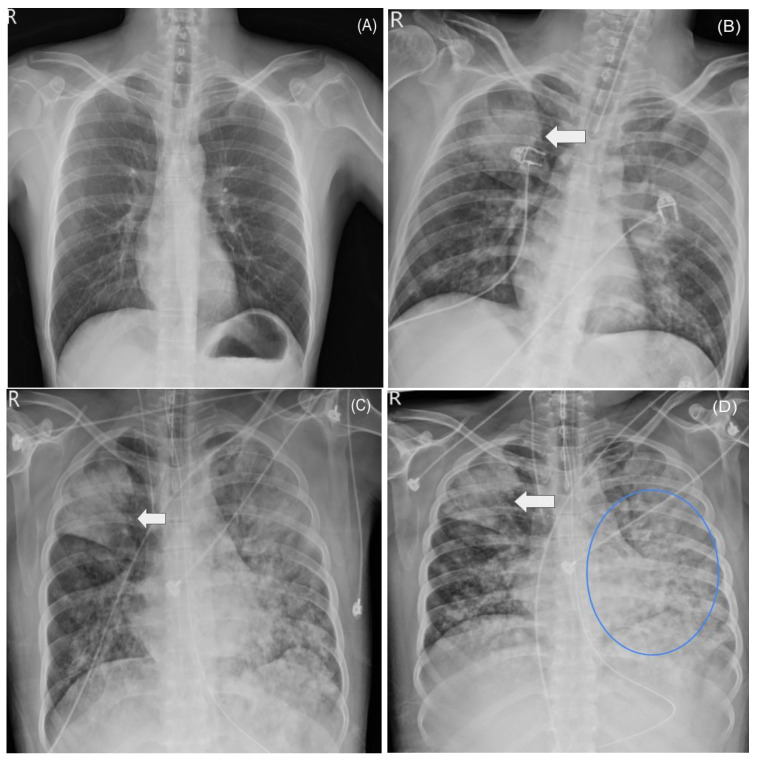
Radiographic progression of pneumonia and ARDS. Radiographic evolution of bilateral pneumonia and ARDS is illustrated in Figure 1. (**A**) Chest X-ray on 3 April 2025, at initial admission: Clear lung fields without infiltrates. (**B**) Chest X-ray on 5 April 2025, after clinical deterioration and ICU transfer: New bilateral pulmonary infiltrates. Arrow indicates initial pneumonia-related opacity. (**C**) Chest X-ray on 7 April 2025, during ARDS progression: Marked increase in bilateral diffuse opacities. Arrow indicates region of consolidative change related to severe pneumonia. (**D**) Chest X-ray on 10 April 2025, terminal phase: Persistent, severe bilateral consolidation. Arrow highlights persistent pneumonia opacity; blue ellipse marks extensive new opacities involving the right lower lung field.

A summarized timeline of the patient’s ICU course, including organ support and CytoSorb use, is shown in Figure 1. Due to progressive respiratory failure (SpO_2_ 48% on mask, PaO_2_ 48 mmHg, PaO_2_/FiO_2_ ratio 60), he was transferred to the intensive care unit (ICU) on 5 April 2025. He underwent endotracheal intubation and mechanical ventilation (FiO_2_ 100%, positive end-expiratory pressure [PEEP] 18 cmH_2_O), with subsequent prone positioning. Chest radiographs and computed tomography (CT) scans demonstrated evolving bilateral pneumonia and acute respiratory distress syndrome (ARDS). A timeline of major chest imaging findings is presented in Figure 2.

### 2.4. Infection Control and Antimicrobial Management

Serial microbiological testing revealed persistent CRPA in blood and bronchoalveolar lavage (BAL) cultures. Microbiological testing and susceptibility determination were performed using the VITEK 2 Compact system (bioMérieux, Marcy-l’Étoile, France) in accordance with the Clinical and Laboratory Standards Institute (CLSI) 2024 guidelines. Minimum inhibitory concentrations (MICs) were interpreted based on CLSI breakpoints. Carbapenemase genotyping was not available at our center during the study period, and molecular characterization could not be performed due to time and resource constraints amid the patient’s fulminant clinical deterioration. Co-isolated methicillin-resistant coagulase-negative staphylococci (MR-CNS) from central venous catheter tip culture were considered catheter colonization rather than bloodstream infection, as repeated blood cultures remained dominated by carbapenem-resistant *P. aeruginosa* (CRPA). The full microbiology timeline is shown in Table 1. Despite escalation to broad-spectrum antibiotics—including meropenem, amikacin, isavuconazole, teicoplanin, and ceftazidime-avibactam (CAZ/AVI)—the infection remained uncontrolled. Colistin was added based on susceptibility testing (MIC 0.5 mg/L, other agents resistant). Antimicrobial Rationale (salvage combination). Given that colistin was the only agent with confirmed in vitro activity (MIC 0.5 mg/L) and the carbapenemase mechanism of the CRPA isolate was not genotyped at our center, a salvage combination of colistin plus ceftazidime–avibactam (CAZ/AVI) was initiated under refractory shock. This choice aimed to maximize potential coverage against non-MBL mechanisms while acknowledging heterogeneous evidence and nephrotoxicity risks. CAZ/AVI was administered from 5 April to 8 April, and colistin from 5 April until death (10 April). This regimen is context-bound and should not be interpreted as a general recommendation.

**Table 2:** Antibiotic timeline, dosage, indication, and microbiology. Abbreviations: CRPA, carbapenem-resistant *P. aeruginosa*; MR-CNS, methicillin-resistant coagulase-negative staphylococci; IV, intravenous; PO, oral administration; MIC, minimum inhibitory concentration; LD, loading dose; qd, once daily; q8h, every 8 h; q12h, every 12 h; S, susceptible; R, resistant; FN, febrile neutropenia; XDR, extensively drug-resistant.

Drug (Generic Name)	Start Date	Stop Date	Dosage	Route	Indication	Microbiology Result (MIC)	Rationale/Notes
Piperacillin/Tazobactam	3 April 2025	4 April 2025	4.5 g q8h	IV	Empiric	CRPA R (≥16)/*Staph.* spp. +	Initial FN, stopped after CRPA ID
Fluconazole	3 April 2025	5 April 2025	200 mg qd	PO	Empiric	—	Added for possible fungal infection
Brosym (Cefoperazone/Sulbactam)	4 April 2025	5 April 2025	2 g q12h	IV	Escalation	—	Deterioration under initial therapy
Meropenem	5 April 2025	6 April 2025	500 mg q8h	IV	Escalation	CRPA R (≥8)	Severe sepsis/respiratory failure
Colistin	5 April 2025	10 April 2025	9 MIU LD, 4.5 MIU q12h	IV	Targeted	CRPA S (MIC 0.5)	Combined with CAZ/AVI salvage
Ceftazidime–Avibactam	5 April 2025	8 April 2025	2.5 g q8h	IV	Targeted	CRPA R (—)	Institutional salvage protocol/XDR
Amikacin	6 April 2025	10 April 2025	250 mg qd	IV	Escalation	CRPA R (≥64)	Added for double coverage
Teicoplanin	6 April 2025	10 April 2025	200 mg q12h → qd	IV	Gram +	MR-CNS S	For catheter/skin coinfection
Isavuconazole	6 April 2025	10 April 2025	200 mg q8h → qd	IV	Empiric	—	Added for suspected fungal infection

Note: Start/stop dates correspond to actual hospital course. Susceptibility based on blood or BAL culture as indicated. Doses per institutional protocol. Ceftazidime–avibactam susceptibility was not available; CAZ/AVI was administered 5–8 April as salvage alongside colistin (5–10 April). Targeted-therapy detail: colistin susceptible (MIC 0.5 mg/L); CAZ/AVI susceptibility not available; CAZ/AVI 5–8 April, colistin 5–10 April. Alternative tetracycline-based combinations were considered but not used in accordance with the center’s rescue protocol. + indicates a positive culture result.

**Table 3:** Major ICU interventions: ventilation, continuous veno-venous hemofiltration (CVVH), prone positioning, and CytoSorb™—timing and duration. Abbreviations: ICU, intensive care unit; CVVH, continuous veno-venous hemofiltration, ARDS, acute respiratory distress syndrome; BAL, bronchoalveolar lavage; CRRT, continuous renal replacement therapy; CVVH, continuous veno-venous hemofiltration; ICU, intensive care unit.

ICU Day	Date	Mechanical Ventilation	Prone Position	CWH (CRRT)	CytoSorb™	Vasopressor	Notes
1	5 April 2025	Start				Start	Intubation, ICU admission
2	6 April 2025	Yes	Start	Start	Start	Yes	Severe shock, ARDS diagnosis
3	7 April 2025	Yes	Yes	Yes	End	Yes	BAL performed. persistent ARDS
4	8 April 2025	Yes		Yes		Yes	
5	9 April 2025	Yes		Yes		Yes	
6	10 April 2025	Yes (to death)		End (death)		Yes	Death (refractory shock/ARDS)

Note: Duration reflects continuous days of intervention. Timing is presented as days since ICU admission.

Alternative regimens considered. In parallel, alternative tetracycline-based salvage backbones (e.g., colistin plus tigecycline/minocycline) were considered in line with prior reports; however, given the patient’s fulminant course, local rescue protocol prioritizing a β-lactam/β-lactamase inhibitor partner, and colistin being the only confirmed active agent, we selected colistin plus CAZ/AVI for time-critical escalation in this XDR phenotype. We do not infer superiority over tetracycline-based combinations; this choice reflects pragmatic, center-specific salvage practice under diagnostic uncertainty.

Susceptibility profile and course. All CRPA isolates were resistant to β-lactams and aminoglycosides and susceptible to colistin (MIC 0.5 mg/L); ceftazidime–avibactam susceptibility testing was not available at our center. CAZ/AVI was administered 5–8 April as salvage, and colistin 5–10 April.

### 2.5. Supportive Care and Clinical Course

The patient’s course was further complicated by refractory shock and multi-organ dysfunction. Continuous renal replacement therapy (CRRT) and early adjunctive CytoSorb™ hemoadsorption were initiated on 6 April due to worsening metabolic acidosis and rising inflammatory markers. Despite transient hemodynamic improvement after CytoSorb therapy, the patient continued to require high-dose vasopressor support, and hematologic recovery did not occur (absolute neutrophil count < 0.01 × 10^3^/μL). Serial chest radiographs demonstrated persistent, severe bilateral infiltrates consistent with ARDS. No cytokine assays (IL-6, TNF-α, or others) were obtained during the ICU course; therefore, any perceived hemodynamic or biochemical changes cannot be linked to cytokine removal mechanistically.

### 2.6. Outcome

Despite maximal supportive therapy—including mechanical ventilation, prone positioning, broad-spectrum antimicrobials, CRRT, CytoSorb, corticosteroids, and transfusional support—there was no meaningful clinical improvement. The patient expired on 10 April 2025, from refractory septic shock and severe ARDS in the context of terminal, treatment-refractory AML.

## 3. Results

### 3.1. Clinical Course

**Figure 3:**
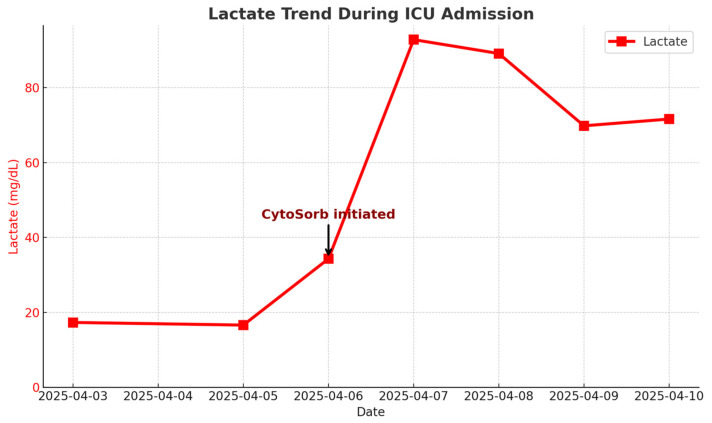
Serial lactate levels during ICU admission. Lactate (mg/dL) is shown as a red line with square markers. The red arrow indicates the initiation of CytoSorb hemoadsorption. Note: Cytokine levels (e.g., IL-6, TNF-α) were not measured; therefore, no mechanistic conclusions regarding cytokine adsorption or immunomodulation can be drawn. The arrow denotes timing only and does not imply causality.

Serum lactate peaked at 34.3 mg/dL (3.8 mmol/L) before CytoSorb initiation. Following CytoSorb therapy and ongoing CVVH, lactate levels declined but remained elevated (>7 mmol/L) throughout the ICU course (Figure 3). After CytoSorb, norepinephrine and vasopressin could be partially weaned, and inflammatory markers (CRP, procalcitonin) showed transient improvement (see Table 3 and Figure 3). Despite these changes, the patient remained dependent on high-dose vasopressors and advanced organ support.

The PaO_2_/FiO_2_ ratio improved from 60 to 120 mmHg with prone positioning and CytoSorb, but bilateral pulmonary infiltrates persisted and ARDS did not fully resolve. Serial chest radiographs confirmed persistent severe diffuse infiltrates (see Figure 1).

Serial blood and BAL cultures remained positive for CRPA across the ICU stay, and no microbiological clearance occurred prior to death. These persistent positive cultures support that infection eradication was not achieved despite combination antimicrobial therapy and organ support. All CRPA isolates were resistant to β-lactams and aminoglycosides, and only susceptible to colistin (MIC 0.5 mg/L). The complete microbiology timeline is summarized in Table 1, and the full antibiotic regimen in Table 2. Ceftazidime–avibactam susceptibility testing was not available at our center; CAZ/AVI was used as part of salvage therapy from 5 April to 8 April alongside colistin (5–10 April). This targeted-antibiotic summary allows attribution analyses to separate antimicrobial exposure (agents and durations) from adjunctive hemoadsorption effects. Because cytokine levels (e.g., IL-6, TNF-α) were not measured, it was impossible to determine whether CytoSorb achieved any cytokine removal or immunomodulatory effect. Consequently, all observed hemodynamic and biochemical changes should be regarded as non-mechanistic, multifactorial phenomena rather than attributable to cytokine adsorption. Therefore, all observed hemodynamic or biochemical changes should be regarded as multifactorial and cannot be directly attributed to CytoSorb therapy. Given the concurrent escalation of organ support (CRRT and proning), antimicrobial modifications, and steroid exposure, the post-initiation trends should be regarded as multifactorial rather than effects of CytoSorb per se.

Tetracycline-based combinations (e.g., colistin plus tigecycline/minocycline) were reviewed as alternatives but were not utilized in this case, consistent with the center’s salvage algorithm favoring a β-lactam/β-lactamase inhibitor partner under refractory shock.

A comprehensive timeline of key clinical events, antimicrobial interventions, and major supportive therapies—including dates of fever onset, culture results, antibiotic changes, ICU transfer, intubation, CVVH, CytoSorb therapy, and clinical deterioration—is depicted in Figure 2.

#### Microbiological Outcomes

During the ICU course, serial blood and BAL cultures remained positive for CRPA without any documented negative conversion; therefore, microbiological clearance was not achieved before death. This aligns with the clinical trajectory and reinforces that any transient hemodynamic improvement cannot be attributed to infection eradication.

### 3.2. Hematologic and Organ Support

Despite repeated filgrastim administration, the absolute neutrophil count (ANC) remained persistently <0.01 × 10^3^/μL throughout the ICU stay. Despite repeated filgrastim, the absence of neutrophil recovery argues against neutrophil-mediated infection control during the observed stabilization. Platelet counts dropped to as low as 12 × 10^3^/μL and were refractory to multiple transfusions. No meaningful hematologic recovery occurred prior to death. Renal support with CVVH was intermittently required for anuria and metabolic acidosis.

### 3.3. Outcome

Despite maximal supportive therapy—including escalation of antibiotics, prone ventilation, repeated CVVH, CytoSorb therapy, steroids, and transfusional support—the patient’s clinical status continued to deteriorate.

He died on 10 April 2025, from refractory septic shock and severe ARDS in the context of terminal, treatment-refractory AML.

## 4. Discussion

Microbiological eradication was never achieved in this case, and the patient died within days of CytoSorb initiation despite maximal intensive care support. Therefore, this case provides no evidence of clinical efficacy for CytoSorb in infection control or survival improvement. All hemodynamic or biochemical changes observed after therapy should be interpreted as temporal associations within a multifactorial treatment context. Microbiological eradication was not achieved, and the patient died within days of CytoSorb initiation despite maximal intensive care support. This case therefore provides no evidence of clinical efficacy for CytoSorb in either infection control or survival improvement. All observed hemodynamic and biochemical changes should be interpreted as non-causal, multifactorial associations within a complex treatment context. This case highlights several critical learning points in managing CRPA sepsis in neutropenic AML patients:

(1) persistent culture positivity despite early aggressive therapy underscores the limited efficacy of current antibiotics;
(2) early CytoSorb use may offer transient hemodynamic stabilization but not definitive clinical benefit [9,10]; and
(3) profound neutropenia remains the key barrier to infection control [11,12].

The increasing prevalence of carbapenem-resistant *P. aeruginosa* among immunocompromised hosts continues to pose major therapeutic challenges [13].

New antibiotic options have emerged for resistant strains, including ceftazidime–avibactam [14].

Cefiderocol has been explored as a potential alternative for XDR phenotypes [15].

However, despite these advances, outcomes in immunocompromised hosts remain poor [16]. While early administration of active agents remains essential, clinical outcomes are often determined by host immunity and infection control rather than antimicrobial potency alone.

Adjunctive hemoadsorption therapy such as CytoSorb has been explored for cytokine modulation in septic shock, but available evidence remains inconclusive. Recent meta-analyses show transient hemodynamic improvements without consistent survival benefit [17,18,19]. In our case, transient lactate and vasopressor changes should therefore be interpreted as non-causal temporal associations rather than as evidence of efficacy.

### 4.1. CRPA Sepsis and Current Treatment Paradigm

The increasing prevalence of CRPA among immunocompromised hosts is associated with high morbidity and prolonged ICU stays [13,14]. Timely administration of effective antibiotics and source control are central to management [15]. However, available antibiotic options are severely limited due to multidrug resistance, and the selection of salvage regimens—such as colistin, aminoglycosides, and ceftazidime-avibactam (CAZ/AVI)—relies on local susceptibility patterns and clinical urgency [16,20,21]. Our use of a colistin plus CAZ/AVI combination was based on in vitro susceptibility and recent salvage protocols for XDR *Pseudomonas* [22,23], though clinical evidence for this regimen remains mixed, with meta-analyses showing variable outcomes and significant nephrotoxicity risks [24,25].

In this case, CAZ/AVI was selected pragmatically as a non-standard salvage partner to colistin under diagnostic uncertainty (no genotypic mechanism identified) and fulminant physiology. We do not claim superiority over alternatives (e.g., colistin–tetracycline backbones); rather, the choice reflects a time-critical, mechanism-agnostic attempt to broaden potential activity in an XDR phenotype where colistin was the sole confirmed active. Any interpretation of benefit remains strictly non-causal given the persistent culture positivity and multiple concomitant interventions.

Alternative tetracycline-based salvage regimens have been reported with variable outcomes and significant toxicity concerns. In our case, these were not employed due to the fulminant course and institutional protocol prioritizing β-lactam-based combinations.

### 4.2. Rationale and Evidence for CytoSorb™ Hemoadsorption

The use of CytoSorb™ as an adjunct in septic shock is predicated on the hypothesis that cytokine removal may modulate the dysregulated host inflammatory response (the so-called “cytokine storm”) [17,18]. Preclinical studies and small observational trials have suggested potential benefits, such as reduction in vasopressor requirements and selected inflammatory biomarkers [19,26]. However, large randomized controlled trials have not demonstrated a consistent survival benefit, particularly in heterogenous ICU populations [27,28]. In the most recent meta-analysis, CytoSorb was associated with transient hemodynamic stabilization but not with lower ICU mortality [29]. International guidelines—including the Surviving Sepsis Campaign 2021 update—do not recommend routine hemoadsorption outside of clinical trials or highly selected cases [30].

Mechanistic uncertainty in the present case. Cytokine monitoring was not performed, and statements about CytoSorb’s putative mechanism were reframed as hypothesis-generating rather than causal. Accordingly, we interpret the observed hemodynamic and biomarker changes as non-causal temporal associations within a multi-intervention context.

In our patient, CytoSorb therapy was initiated early—guided by maximal CytoScore and refractory shock—yet only transient improvements in lactate and vasopressor need were observed, without sustained clinical recovery. This observation is in line with recent real-world series [31,32].

### 4.3. Study Limitations and Gaps

Several limitations of this report must be acknowledged:

Microbiological clearance was not achieved, and follow-up cultures were persistently positive. This precluded any mechanistic inference regarding the efficacy of source control or CytoSorb in infection eradication [33].

First, mechanistic inference is not possible: cytokine assays were not obtained. This absence of cytokine profiling fundamentally limits any mechanistic attribution of observed hemodynamic or biochemical trends to CytoSorb therapy; all interpretations must remain descriptive and hypothesis-generating only. No cytokine level (IL-6, TNF-α, etc.) measurements were performed due to logistical and resource constraints. Thus, the central pathophysiological hypothesis (cytokine storm mitigation) cannot be confirmed in this case. As such, any interpretation regarding cytokine modulation or immunologic impact of CytoSorb in this case would be speculative and unsupported by biochemical evidence. Genotypic characterization of the carbapenemase mechanism (e.g., blaVIM, blaIMP, blaNDM) was not performed due to the lack of rapid molecular testing capacity and the patient’s rapid clinical decline. This remains an important limitation when interpreting antimicrobial response in XDR. *P. aeruginosa* infections.

Multiple concurrent interventions were administered—including aggressive antibiotics, CVVH, steroids, filgrastim, and prone positioning—rendering any causal attribution to CytoSorb highly speculative.

The patient remained persistently neutropenic despite repeated filgrastim, a key factor likely precluding infection control and hematologic recovery.

As with all single-case reports, generalizability is limited by the unique AML cytogenetic background, disease severity, and ICU resource availability. Furthermore, the patient’s unique cytogenetic profile—acute myeloid leukemia with inv(3)(q21q26.2) and monosomy 7—represents a rare and aggressive subtype associated with poor prognosis and profound treatment resistance. This genetic background, together with the single-center nature of this observation, limits the external generalizability of our findings. Therefore, this report should be regarded primarily as a descriptive clinical observation that underscores the challenges of infection management in highly immunocompromised hosts rather than as evidence supporting any novel therapeutic efficacy of CytoSorb.

### 4.4. Why Certain Measures Were Not Done

Certain key investigations, including serial cytokine monitoring and rapid molecular diagnostic assays, were not feasible in this real-world scenario due to cost, time constraints, and institutional resource limitations. Similarly, our inability to confirm microbiological clearance (by serial blood/BAL cultures) reflects both the fulminant course and logistical challenges common in overwhelmed ICU settings [34,35].

### 4.5. Clinical and Research Implications

Despite the lack of microbiological eradication and the failure to reverse shock, the case underscores several practice points:

CytoSorb may have a role as an adjunct for temporary hemodynamic stabilization, but cannot substitute for effective antibiotics or definitive source control [36,37]. For CRPA infections, rigorous infection source control (e.g., timely device removal or drainage when feasible) and optimization of active antimicrobials remain the primary drivers of outcome; adjunctive hemoadsorption cannot compensate for failure to eradicate the pathogen.

In profoundly immunosuppressed patients (such as AML with persistent neutropenia), infection control remains the primary determinant of outcome.

Biomarker-guided therapy and early cytokine monitoring may help clarify which subgroups could benefit from adjunctive therapies.

Future research should focus on prospective, multicenter trials examining hemoadsorption in narrowly defined patient populations, with mechanistic endpoints such as cytokine clearance and microbial eradication [16,18,25].

### 4.6. Attribution and Confounding

In this case, transient reductions in lactate and vasopressor requirements occurred concurrently with antimicrobial escalation, CRRT, prone ventilation, and steroid exposure, while ANC failed to recover despite filgrastim. Together with persistent CRPA-positive cultures, these features preclude causal inference that favors CytoSorb. All changes were multifactorial and cannot be attributed to CytoSorb alone. Accordingly, this report should be regarded as hypothesis-generating and not as evidence of device efficacy. Standard intensive care measures, including evidence-based ventilation strategies, prone positioning, lung-protective mechanical ventilation, extracorporeal support for refractory hypoxemia, adherence to sepsis definitions and guidelines, timely antimicrobial optimization, and the use of corticosteroids in septic shock, were followed in accordance with established recommendations.

## 5. Conclusions

Early adjunctive CytoSorb may offer transient clinical stabilization in select patients with refractory septic shock and ARDS, but its effect cannot be isolated from the impact of antimicrobial therapy, organ support, and host factors. Infection source control and microbiological eradication remain central to the management of CRPA sepsis. CytoSorb should be considered only as part of a multimodal approach, and not a substitute for standard-of-care interventions. Any observed stabilization should be ascribed to bundled critical-care measures rather than to CytoSorb alone, given overlapping co-interventions and absent microbiological clearance. As a single-case report with persistent culture positivity and a fatal outcome, these observations do not support efficacy claims for CytoSorb and should be interpreted as hypothesis-generating only. Larger, controlled studies are required to clarify its role. Further extended discussion and supporting literature are provided in Appendix A.

## Figures and Tables

**Table 1:** Microbiology timeline: all cultures, results, dates. Abbreviations: CRPA, carbapenem-resistant *P. aeruginosa*; *Staph.* spp., Staphylococcus species; MR-CNS, methicillin-resistant coagulase-negative staphylococci; CVC, central venous catheter; BAL, bronchoalveolar lavage; S, susceptible; R, resistant; MIC, minimum inhibitory concentration; AFB, acid-fast bacilli. All susceptibility testing was performed using the VITEK 2 Compact platform according to CLSI 2024 interpretive standards.

Date	Sample	Pathogen/Method	Result	Sensitivity/MIC
3 April	Blood	CRPA, *Staph.* spp.	Positive	Colistin S (MIC 0.5), others R
4 April	CVC tip	MR-CNS	Positive	
5 April	BAL	CRPA	Positive	Same as above
6 April–9 April	Sputum	CRPA	Positive	
7 April	BAL	Culture, FilmArray: CRPA	Positive	
6 April	Fungus, CVC	Negative	Negative	
6 April	Virus, Blood	Not Detected	Negative	
7 April	BAL	Gram-stain, AFB, TB, Fungus	Negative	
8 April	Sputum	CRPA	Positive	
8 April	Sputum	TB, Fungus	Negative	
8 April	Blood	CRPA	Positive	

Note: Dates are presented as 2025/MM/DD unless otherwise specified. All *Pseudomonas* isolates were resistant to all β-lactams except colistin (see main text/Table 2 for detailed antibiotic timeline and susceptibility data). All follow-up blood and BAL cultures remained positive for CRPA; no negative cultures were documented before death. Susceptibility summary: CRPA isolates *colistin-susceptible* (MIC 0.5 mg/L); β-lactams/aminoglycosides resistant; CAZ/AVI susceptibility not available (see Table 3 for agent-level dosing and dates).

## Data Availability

The original contributions presented in this study are included in the article/Supplementary Materials. Further inquiries can be directed to the corresponding author.

## Supplementary Materials

The following supporting information can be downloaded at: https://www.mdpi.com/article/10.3390/diseases13120382/s1, Table S1. Chemotherapy regimens and dosages.

## Appendix A

In this case, persistent culture positivity confirmed that infection eradication was not achieved; accordingly, any transient stabilization should not be interpreted as evidence of pathogen clearance or definitive therapeutic effect.

**Appendix Discussion**

This Appendix note provides additional context and literature support extending the main discussion.

Recent international guidelines emphasize that the timing of hemoadsorption initiation and appropriate patient selection are likely critical to maximizing potential benefit.

Preclinical and clinical studies have shown that CytoSorb can reduce circulating cytokines and selected inflammatory biomarkers, although survival benefit remains unproven in randomized trials.

Reports on colistin–tetracycline combinations show heterogeneous outcomes and toxicity profiles across different settings.
