# Supplementary material for: Early CytoSorb Hemoadsorption in a Neutropenic Acute Myeloid Leukemia Patient with Carbapenem-Resistant Pseudomonas Septic Shock and ARDS
Source: Diseases. 2025 Nov 24;13(12):382. doi: 10.3390/diseases13120382 (PMC12732260; doi:10.3390/diseases13120382)
Supplement: Supplementary file 1 [file diseases-13-00382-s001.zip › diseases-3889839-supplementary.pdf]

Table S1. Chemotherapy regimens and dosages.

Cycle	Dates	Regimen	Drugs/Dose	Route/Schedule
Induction	2025/01/24–31	I3A7	Cytarabine, Daunorubicin	IV, standard
Consolidation	2025/03/25–29	Cytarabine	4680 mg (500 mg/vial)	IV, Q12H
Targeted	2025/02/04–	Venetoclax	100 mg QD	PO
